# Sex differences in cerebral infarction in Norway: Analysis of data from the Norwegian Stroke Registry 2018–2022

**DOI:** 10.1093/esj/23969873251383322

**Published:** 2026-01-01

**Authors:** Ingrid Helene Engås, Torunn Varmdal, Hanne Ellekjær

**Affiliations:** Department of Neuromedicine and Movement Science, Faculty of Medicine and Health Sciences, NTNU – Norwegian University of Science and Technology, Trondheim, Norway; Department of Medical Quality Registries, St. Olav’s Hospital, Trondheim University Hospital, Trondheim, Norway; Department of Circulation and Medical Imaging, Faculty of Medicine and Health Science, NTNU- Norwegian University of Science and Technology, Trondheim, Norway; Department of Neuromedicine and Movement Science, Faculty of Medicine and Health Sciences, NTNU – Norwegian University of Science and Technology, Trondheim, Norway; Department of Stroke, Clinic of Medicine, St. Olavs Hospital, Trondheim University Hospital, Trondheim, Norway

**Keywords:** Stroke cerebral infarction brain infarction ischemic stroke sex differences sex disparity sex characteristics epidemiology observational study time-to-treatment neurologic manifestations

## Abstract

**Introduction:**

Whether sex influence the clinical pathway of stroke, has been debated. In this study, we want to compare and add knowledge to presentation of symptoms and time to treatment of cerebral infarction in men and women.

**Patients and methods:**

Data on 38,489 patients with cerebral infarction from 2018 to 2022 were obtained from the Norwegian Stroke Registry (NHR). The analyses were stratified by sex and age groups.

**Results:**

Overall, there was a substantial sex parity for both focal neurological deficits and time to treatment. However, women were less likely to be awake at admission (RR 0.96, CI 0.95–0.97). A higher proportion of women presented with FAST symptoms (RR 1.06, CI 1.04–1.07), and women presented more often with arm paresis (RR 1.11, CI 1.08–1.14), facial paresis (RR 1.08, CI 1.05–1.11), aphasia (RR 1.17, CI 1.13–1.21), leg paresis (RR 1.18, CI 1.15–1.21) and neglect (RR 1.24, CI 1.18–1.31). Men presented more often with ataxia (RR 0.85, CI 0.81–0.89) and double vision (RR 0.72, CI 0.64–0.81). At admission, women had significantly higher National Institutes of Health Stroke Scale (NIHSS) score compared to men (average 5.60 vs 4.66), and significantly longer time from symptom onset to notification of Emergency Medical Communication Center (EMCC; average 298 vs 282 min)

**Discussion and conclusion:**

Our findings indicate a large degree of sex equality in terms of symptoms and time to treatment for Norwegian patients with cerebral infarction. Further research exploring possible sex disparity in stroke treatment, is warranted.

## Graphical Abstract

**Graphical Abstract:**
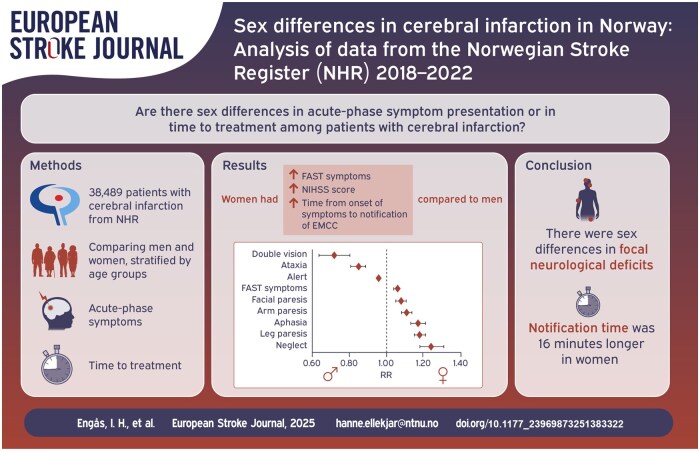

## Introduction

In Norway, 11,084 patients were hospitalized with acute stroke in 2022,^1^ and nearly 9 out of 10 of them had a cerebral infarction.^2^ Treatment options, and thus the outcomes of a cerebral infarction, are largely determined by prompt and accurate diagnosis.^3^ The patient’s immediate recognition of stroke symptoms, notification, and further correct assessment and diagnosis in the emergency room, are determinants of a successful outcome. Although stroke largely affects both sexes, there has been limited research on sex differences in the patient care pathway.

It is known that diseases can present and progress differently depending on whether the individual is a man or a woman. The existing literature is divided on whether sex influences the clinical pathway of stroke. As to focal neurological deficits, previous research shows a high degree of similarity, but also possible differences to a varying degree. Some of the findings are even contradictory.^4–9^ Previous research is also inconclusive about sex differences in severity.^9–16^ However, several studies have shown that women tend to present with lower level of consciousness, and possibly a higher NIHSS score than men.^4–7,14–18^ Recent systematic reviews suggests that women experiencing stroke may present with more generalized symptoms like headache, confusion, incontinence, fatigue, nonspecific weakness or pain, seizures, and convulsions. However, the literature is not unanimous on this matter.^4–8^ Findings are more consistent regarding sex equality in time from symptom onset to admission, and admission to reperfusion. Still, some studies indicate a potential notification delay in women.^11,17–22^

## Patients and methods

### Study design and study population

This is a descriptive cohort study using data from the NHR for the period 2018–2022. A total of 38,489 patients diagnosed with cerebral infarction (ICD-10 code I63), admitted to Norwegian hospitals within 28 days from onset, were included. NHR has registered patients over the age of 18 with acute stroke as part of the National Register of Cardiovascular Diseases since 2012. Acute phase symptoms are defined as symptoms and findings at admission or within 24 h after admission. It is mandatory for all Norwegian hospitals to report without patient consent, which ensures a substantial and representative dataset. According to the 2022 annual report, the coverage was 89%. Studies on NHR’s data quality have shown that the registry is largely valid and reliable.^2,23–25^

### Statistical analysis

Analyzes were performed using the Statistical Package for the Social Sciences (SPSS, version 29). The study population was stratified by sex and age groups. For categorical variables, differences between groups were analyzed using the chi-square test, with frequencies, *p*-values, and relative risk (RR) with 95% confidence intervals (CI). RR and corresponding CIs were calculated using MedCalc software (version 22.023). For continuous variables, group differences were analyzed with t-test, and results are presented with *p*-values, means, standard deviations, and medians. Negative values and outliers, defined as z-scores greater or less than ±3, were excluded from the time analyses. A two-sided significance level of 5% (*p* < 0.05) was applied to all statistical tests.

## Ethics

The Regional Committee for Medical and Health Research Ethics in Norway (REC number 584387) approved the study.

## Results

### Patient characteristics

Median age was 74 years for men, and 79 years for women. Men accounted for 55% of the total study population, and there was a predominance of men in all age groups, except ⩾ 85 years. Prestroke, a larger proportion of men lived at home without home healthcare services and had a partner. Men also had a higher functional level before the stroke compared to women. Equal proportions of men and women received thrombolysis. The proportion of women who underwent thrombectomy was slightly higher than for men, but non-significant (Table 1).

**Table 1.** Patient characteristics by sex (*N* = 38,489).

Characteristics	Total	Women	Men	*p*-value
Patients *N*, women/men *n* (%)	38,489	17,063 (44.3)	21,426 (55.7)	
Age at admission (years), mean (SD)				<0.001*
Age (years), mean (SD)	74.0 (12.89)	76.5 (13.00)	72.0 (12.44)	<0.001*
Age (years), median (min, max)	76 (18, 107)	79 (18, 107)	74 (18, 102)	
18–44 years, *n*	983	437	546	
45–54 years, *n*	2219	750	1469	
55–64 years, *n*	4779	1477	3302	
65–74 years, *n*	9758	3609	6149	
75–84 years, *n*	12,397	5721	6676	
85+ years, *n*	8353	5069	3284	
Living situation pre stroke (*N* = 38,264)				<0.001*
Lives at home without home healthcare services %	76.0	68.1	82.3	
Lives at home with home healthcare services %	16.1	20.8	12.4	
Lives in assisted living or nursing home %	7.9	11.0	5.3	
Marital status pre stroke (*N* = 37,066)				23969873251383322<0.001*
Married/partner %	57.5	42.3	69.3	
Widow/widower %	20.4	34.6	9.5	
Single %	22.1	23.1	21.2	
Mobility pre stroke (*N* = 37,908)				23969873251383322<0.001*
Alone outdoors and indoors %	87.2	83.0	90.6	
Alone indoors %	8.3	11.2	6.0	
Assisted %	4.5	5.8	3.4	
Pre-stroke modified Rankin Scale (mRS; *N* = 35,192)				23969873251383322<0.001*
No symptoms (mRS 0) %	54.6	48.6	59.2	
No significant disability (mRS 1) %	18.8	19.1	18.6	
Slight disability (mRS 2) %	13.0	14.8	11.6	
Moderate disability (mRS 3) %	9.2	11.6	7.2	
Moderate severe disability (mRS 4) %	3.9	5.0	3.1	
Severe disability (mRS 5) %	0.5	0.8	0.3	
Treatment given (*N* = 38,489)				
Thrombolysis, *n* (%)	7996 (20.8%)	3531 (20.7%)	4465 (20.8%)	0.630
Thrombectomy, *n* (%)	2224 (5.8%)	1033 (6.1%)	1191 (5.6%)	0.142

SD: standard deviation.

^*^Significant sex difference (*p* < 0.05).

## Symptoms in the acute phase

### Level of consciousness

Upon admission, women were less likely to be alert compared to men (RR 0.96, *p* < 0.001). This was most evident among patients ⩾ 85 years, where 81,0% of women compared to 85,8% of men were alert on admission. There were no significant sex differences in alert patients < 75 years. (Table 2, Supplemental Table I)

**Table 2.** Level of consciousness upon admission for men and women (*N* = 38,399).

Level of consciousness	Total (%)	Women (%)	Men (%)	RR (95% CI)	*p*-value
Alert; keenly responsive	89.2	87.2	90.9	0.96 (0.95–0.97)	<0.001*
Not alert, but arousable by minor stimulation	6.3	7.5	5.3	1.40 (1.30–1.51)	<0.001*
Not alert; requires repeated stimulation	2.2	2.9	1.7	1.67 (1.46–1.90)	<0.001*
Unresponsive or responds only with reflex	1.6	1.8	1.3	1.35 (1.15–1.59)	<0.001*
Unknown	0.7	0.7	0.7	0.93 (0.74–1.18)	0.030

CI: confidence interval; RR: relative risk for women versus men.

^*^Significant sex difference (*p* < 0.05).

### Focal deficits

There were no sex differences in prevalence of dysarthria, sensory deficits, visual field deficits, vertigo, or dysphagia. Women presented with arm paresis, facial palsy, aphasia, leg paresis, and neglect to a significant greater extent than men. Men presented with ataxia and double vision to a significant greater extent than women. Age-stratified analysis revealed that arm paresis was the most frequently occurring symptom across all age groups and in both sexes. Overall, sex differences tended to increase with age, and the total symptom burden increased with age for most focal deficits in both sexes. Exceptions were sensory deficits, vertigo, and double vision, which occurred more frequently in patients aged 18–44, compared to those aged ⩾ 85 in both sexes. (Table 3, Supplemental Tables IIa and IIb).

**Table 3.** Focal deficits for men and women.

Focal deficits	Total (%)	Women (%)	Men (%)	RR (95% CI)	*p*-value
Arm paresis (*N* = 38,399)	42.2	44.7	40.1	1.11 (1.08–1.14)	<0.001*
Facial palsy (*N* = 38,399)	38.2	39.9	37.0	1.08 (1.05–1.11)	<0.001*
Leg paresis (*N* = 38,399)	38.0	41.5	35.1	1.18 (1.15–1.21)	<0.001*
Dysarthria (*N* = 38,399)	29.3	29.3	29.4	1.00 (0.97–1.03)	0.870
Aphasia (*N* = 38,398)	28.6	31.2	26.6	1.17 (1.13–1.21)	<0.001*
Sensory deficits (*N* = 38,399)	20.3	19.9	20.6	0.96 (0.93–1.00)	0.073
Ataxia (*N* = 38,399)	16.0	14.5	17.1	0.85 (0.81–0.89)	<0.001*
Visual field deficits (*N* = 38,399)	14.0	14.2	13.9	1.02 (0.97–1.07)	0.408
Neglect (*N* = 38,399)	11.8	13.2	10.6	1.24 (1.18–1.31)	<0.001*
Vertigo (*N* = 38,399)	8.9	8.7	9.2	0.95 (0.89–1.01)	0.100
Dysphagia (*N* = 30,833)	3.5	3.6	3.4	1.06 (0.94–1.20)	0.308
Double vision (*N* = 38,399)	3.2	2.6	3.7	0.72 (0.64–0.81)	<0.001*

CI: confidence interval; RR: relative risk for women versus men.

^*^Significant sex difference (*p* < 0.05).

Our data show that the majority of patients with cerebral infarction, both men and women, presented with one or more FAST symptom at admission (67.4% of men and 71.3% of women). For all age groups combined, as well as within the 65–74 and 85+ age groups, women were significantly more likely than men to present with FAST symptoms. The lowest proportion of FAST symptoms were seen in patients aged 18–44 years, with 56.6% and 59.2% for men and women, respectively (Table 4, Supplemental Table IIa).

**Table 4.** Proportion of women and men with at least one FAST symptom** by age groups (*N* = 38,398).

Age groups	Total (%)	Women (%)	Men (%)	RR (95% CI)	*p*-value
All age groups	69.1	71.3	67.4	1.06 (1.04–1.07)	<0.001*
18–44 years	57.8	59.2	56.6	1.05 (0.94–1.16)	0.412
45–54 years	60.3	59.5	60.7	0.98 (0.91–1.05)	0.595
55–64 years	63.6	65.3	62.9	1.04 (0.99–1.09)	0.102
65–74 years	66.1	67.6	65.2	1.04 (1.01–1.07)	0.015*
75–84 years	70.5	71.1	70.0	1.02 (0.99–1.04)	0.195
85+ years	77.4	78.7	75.4	1.04 (1.02–1.07)	<0.001*

CI: confidence interval; RR: relative risk for women versus men.

^*^Significant sex difference (*p* < 0.05).

^**^FAST symptoms: Facial palsy, arm paresis, aphasia and dysarthria.

## NIHSS-score

Overall, there was a significant sex difference in stroke severity, measured by NIHSS at admission, with women scoring on average approximately one point higher than men (5.60 vs 4.66). The difference was significant in women above 65 years of age (Table 5).

**Table 5.** NIHSS score at admission for men and women by age groups (*N* = 32,125).

Age groups	Women				Men				23969873251383322*p*-value
	Mean (SD)	Median	Min.	Max.	Mean (SD)	Median	Min.	Max.	
All age groups	5.60 (6.40)	3	0	41	4.66 (5.66)	3	0	40	<0.001*
18–44 years	3.61 (4.71)	2	0	25	3.85 (5.47)	2	0	38	0.514
45–54 years	3.88 (5.07)	2	0	26	3.60 (4.74)	2	0	29	0.242
55–64 years	4.15 (5.62)	2	0	38	4.02 (5.32)	2	0	40	0.484
65–74 years	4.64 (5.75)	3	0	40	4.15 (5.18)	2	0	36	<0.001*
75–84 years	5.48 (6.27)	3	0	41	4.94 (5.80)	3	0	40	<0.001*
85+ years	7.30 (7.11)	5	0	40	6.35 (6.56)	4	0	40	<0.001*

SD: standard deviation.

^*^Significant sex difference (*p* < 0.05).

## Time intervals

Timing data were available for 50%–70% of the patients, with no sex differences in data availability except for time to imaging, where men had 60% and women had 72% coverage. There was significant sex difference in the time from symptom onset to notification of EMCC all ages combined, with an average of 16 min longer notification time in women compared to men (Table 6). Divided into age groups, significant sex difference was only observed among patients from 65 to 74 years, where EMCC on average were notified 36 min later for women. Among the youngest patients aged 18–54, the pattern was reversed, with men having a longer, though non-significant, delay (Table 6, Supplemental Table III). Further stratified analyses showed that regardless of sex, time to notification was significantly longer for patients living alone (346 vs 258 min, *p* < 0.001). Men had significantly longer time from symptom onset to notification of EMCC than women when living alone (336 vs 360 min, *p* = 0.045), while among those not living alone, women had significantly longer notification time than men (269 vs 251 min, *p* = 0.032).

**Table 6.** Time intervals of the cerebral stroke clinical pathway in minutes for men and women.

Time intervals	Women		Men		23969873251383322*p*-value
	Mean (SD)	Median	Mean (SD)	Median	
Symptom onset to EMCC notification (*N* = 18,744)	298.34 (460.08)	94	282.27 (444.38)	85	0.015*
EMCC notification to hospital admission (*N* = 18,887)	63.34 (48.61)	50	64.69 (49.28)	51	0.059
Hospital admission to cerebral CT/MRI (*N* = 25,432)	121.21 (481.74)	28	124.53 (557.95)	27	0.616
Hospital admission to thrombolysis (*N* = 7372)	36.65 (23.74)	30	35.60 (25.07)	28	0.067
Hospital admission to thrombectomy (*N* = 1734)	180.30 (175.29)	135	184.88 (199.18)	135	0.614

SD: standard deviation.

^*^Significant sex difference (p < 0.05).

There were no significant sex differences in time from EMCC notification to hospital admission, or from admission to cerebral CT/MRI, except in the age group 85+, where women underwent imaging on average 23 min earlier than men (Table 6, Supplemental Table III). Time from admission to thrombolysis or thrombectomy, showed no significant sex difference across all ages combined. In age-stratified analyses, significant sex difference was observed only in patients aged 65–74 years, where women had, on average, a 2-min and 40-s longer interval from admission to thrombolysis compared to men (Table 6, Supplemental Table III).

## Discussion

### Key findings

Our data revealed sex parity for time to treatment, and occurrence of dysarthria, sensory deficits, visual field deficits, vertigo and dysphagia. Time from onset of symptoms to notification of EMCC were significantly longer in women. At admission, women had a higher mean NIHSS score than men and were less likely to be alert. Women more frequently presented with FAST symptoms overall, as well as arm paresis, facial paresis, aphasia, leg paresis and neglect when analyzed separately. Men more often presented with ataxia and double vision. Sex differences in stroke severity (NIHSS), level of consciousness, and FAST symptoms were most pronounced in the oldest age groups.

### Implications of our results

Our findings suggest sex specific differences in certain focal deficits, consistent with some previous studies while contradicting others.^4–9^ The observed sex difference in symptom topography, where posterior circulation features appear more prevalent in men and anterior circulation signs more common in women, warrants further investigation. One hypothesis could be different underlying risk profiles. Frid et al.,^26^ found an increased risk of posterior circulation ischemic strokes in diabetic patients and in males after adjusting for conventional risk factors. They suggested that pathogenic processes in the vertebrobasilar arteries may be influenced by genetically determined molecular pathways.

The fact that women were less likely to be awake at admission, aligns with existing literature on stroke.^4–7^ Further exploration of sex differences in infarct volume and localization in addition to notification delay in women, may shed light on this observation. Regardless, our findings, together with previous research, demonstrate a high degree of similarity in focal deficits between men and women.^4–6,8,9^ This study particularly highlights that a majority of both men and women have classical FAST symptoms relevant for all age groups.

Previous research is not conclusive about sex differences in severity.^9–16^ In our study, women had a one point higher mean NIHSS score at admission than men, indicating more pronounced deficits particularly among elderly women. One should bear in mind that the average NIHSS score, and the overall symptom burden increased with age. According to NIHSS, facial palsy, dysarthria, aphasia or neglect, are scored 2–3 points when the patient is comatose. This is relevant as women in our study more often had impaired consciousness on admission. Furthermore, NIHSS has been criticized for being more sensitive to anterior than posterior circulation strokes, as it does not assess tempo, diadochokinesis or balance. Supporting this, Tao et al.^27^ found that signs typically associated with posterior infarctions, such as visual field deficits and oculomotor nerve palsy, were infrequent even in confirmed posterior infarctions.

Our study indicates sex parity in time to treatment for patients with cerebral infraction in Norwegian hospitals. Once the EMCC had been alerted, no sex differences were observed in time from notification to hospital admission or from admission to imaging. This suggests that healthcare workers assess and investigate cerebral infarction in both women and men at comparable speeds. The exception was men over 85 years of age, who underwent imaging later than women. In this age group, men also had a significantly lower prevalence of arm paresis, facial paresis, aphasia, neglect, and leg paresis compared to women. Men were slightly underrepresented in terms of available timing data from admission to imaging. Nevertheless, our analyses showed no sex differences in time from admission to reperfusion with thrombolysis or thrombectomy, consistent with most previous literature.^11,17,19^

Women with cerebral infarction, particularly in the age group 65 and older, had significantly longer time from symptom onset to EMCC notification than men. In our cohort, 69% of men had a spouse or partner, compared to 42% of women, and patients living alone had longer notification times overall. The presence of a household member who can alert emergency services thus appears to be an important factor. Interestingly, among those living alone, men had significantly longer notification time than women, whereas among those living with someone, men had significantly shorter notification time than women. This is a paradox, given that women presented with more severe clinical symptoms. The literature mentions a possible contributing factor, when pointing out that women seem to have more knowledge about warning symptoms of stroke and when to take action.^7,28^ As women often is the bystander for the men and vice versa, does this reveal a knowledge gap among men?

### Strengths and limitations

Our study includes a broad spectrum of patients with cerebral infarction, representing different ages, sexes, geographic regions, functional status, and living situations. Therefore, the study appears to be representative of adult patients with cerebral infarction in Norway. Unlike many of the cited studies, which included only patients receiving reperfusion therapy, our large cohort is broader and may detect clinically relevant differences not observable in smaller or more selective populations. The large dataset enabled precise estimates, and allowed for subgroup analyses, without compromising statistical power. Conducting multiple statistical tests increases the likelihood of chance findings, which may account for some of the observed differences at the age group level. NHR is a registry with high coverage, validity, and reliability. Its systematic data collection and standardized sampling methods help reduce bias and inaccuracy.^23–25^ The data was collected in numerous locations across the country by different healthcare providers. Despite the use of standardized forms, it is likely that individual patient and healthcare provider assessments varied.

The data are representative only of hospitalized patients. Strokes treated in primary care, unrecognized strokes and non-registered hospitalized strokes are not included. The high completeness of registered hospitalized strokes makes it unlikely that selection bias explains our findings. One can argue that some individuals with atypical or diffuse symptoms were never hospitalized and never registered in NHR. However, patients with significant functional decline, or other health problems, would likely require hospital assessment at some point, increasing the probability of eventual registration. All analyses were stratified by sex and age, in line with the study’s objective to explore and quantify sex differences. The study was not designed to investigate the underlying causes of sex differences. For example, we cannot determine whether the notification delay in women was due to patient, bystander or other factors. Confounding variables beyond sex and age remain unanswered.

Recent systematic reviews have associated stroke in women with more generalized symptoms.^4–8^ These symptoms are not available in NHR. Our analyses are limited to focal deficits recorded in NHR, including NIHSS items, vertigo, dysphagia, and double vision. Therefore, we cannot rule out the possibility of sex differences in symptom profile beyond the analyzed symptoms. Still, we cannot find evidence that women are overlooked in the acute phase due to their symptom patterns. Women seem to present with FAST symptoms as often, or more often, as men. If women experience additional global symptoms, it does not seem to impede the healthcare system’s ability to detect and diagnose cerebral infarction.

## Conclusion

Sex parity was documented for both focal neurological deficits and time to treatment. Most patients, regardless of sex, presented with one or more FAST symptom in the acute phase. Women were less likely to be awake at admission, had a one point higher average NIHSS score, and more frequently exhibited arm paresis, facial paresis, aphasia, leg paresis, neglect and FAST symptoms in general. Men presented more often with ataxia and double vision. In terms of time intervals, women had a longer time from symptom onset to notification. Our results highlight that current clinical practice and tools are effective for both sexes. They also underscore the need to develop targeted strategies to ensure timely notification of the EMCC, particularly for elderly women, and individuals living alone. Campaigns should address both men and women, and both future patients, their next of kin and bystanders.

## Supplementary Material

ds-eso_23969873251383322

## Data availability statement

Data from the Norwegian Stroke Registry is accessible through Helsedata at https://helsedata.no, upon request.
