# Supplementary material for: Sex differences in cerebral infarction in Norway: Analysis of data from the Norwegian Stroke Registry 2018–2022
Source: Eur Stroke J. 2026 Jan 1;11(1):23969873251383322. doi: 10.1093/esj/23969873251383322 (PMC12866217; doi:10.1093/esj/23969873251383322)
Supplement: ds-eso_23969873251383322 [file ds-eso_23969873251383322.zip › sj-docx-1-eso-10.1177_23969873251383322.docx]

**Supplemental material**

| Level of Consciousness | Total (%) | Women (%) | Men (%) | RR (95% CI) | p-value |
| --- | --- | --- | --- | --- | --- |
| Alert; keenly responsive | **89,2** | **87,2** | **90,9** | **0.96 (0.95-0.97)** | **<.001*** |
| 18-44 years | 92,6 | 92,0 | 92,3 | 1.00 (0.96-1.04) | .990 |
| 45-54 years | 93,1 | 92,4 | 93,2 | 1.00 (0.97-1.02) | .561 |
| 55-64 years | 92,2 | 90,8 | 92,6 | 0.98 (0.97-1.00) | .098 |
| 65-74 years | 91,6 | 90,7 | 91,9 | 0.99 (0.98-1.00) | .065 |
| 75-84 years | 89,3 | 87,7 | 90,4 | 0.97 (0.96-0.98) | <.001* |
| 85+ years | 83,1 | 81,0 | 85,8 | 0.94 (0.93-0.96) | <.001* |
| Not alert, but arousable by minor stimulation | **6,3** | **7,5** | **5,3** | **1.40 (1.30-1.51)** | **<.001*** |
| 18-44 years | 4,7 | 5,5 | 4,0 | 1.37 (0.78-2.40) | .277 |
| 45-54 years | 3,9 | 3,6 | 4,1 | 0.88 (0.57-1.38) | .583 |
| 55-64 years | 4,5 | 5,5 | 4,0 | 1.38 (1.05-1.80) | .020* |
| 65-74 years | 5,0 | 5,2 | 4,9 | 1.06 (0.88-1.26) | .541 |
| 75-84 years | 6,4 | 7,6 | 5,5 | 1.38 (1.21-1.58) | <.001* |
| 85+ years | 9,3 | 10,3 | 7,9 | 1.30 (1.13-1.50) | <.001* |
| Not alert; requires repeated stimulation | **2,2** | **2,9** | **1,7** | **1.67 (1.46-1.90)** | **<.001*** |
| 18-44 years | 0,8 | 0,7 | 0,9 | 0.75 (0.18-3.13) | .695 |
| 45-54 years | 1,1 | 1,5 | 1,0 | 1.54 (0.70-3.38) | .280 |
| 55-64 years | 1,3 | 1,2 | 1,3 | 0.87 (0.50-1.51) | .615 |
| 65-74 years | 1,4 | 1,9 | 1,1 | 1.66 (1.19-2.31) | .003* |
| 75-84 years | 2,2 | 2,5 | 2,0 | 1.23 (0.97-1.55) | .085 |
| 85+ years | 4,1 | 4,8 | 3,0 | 1.60 (1.28-2.02) | <.001* |
| Unresponsive or responds only with reflex | **1,6** | **1,8** | **1,3** | **1.35 (1.15-1.59)** | **<.001*** |
| 18-44 years | 1,2 | 0,7 | 1,7 | 0,42 (0,11-1.53) | .188 |
| 45-54 years | 0,9 | 1,2 | 0,8 | 1.47 (0.62-3.48) | .379 |
| 55-64 years | 1,2 | 1,2 | 1,3 | 0.91 (0.52-1.59) | .737 |
| 65-74 years | 1,1 | 1,3 | 1,0 | 1.30 (0.89-1.89) | .169 |
| 75-84 years | 1,3 | 1,4 | 1,3 | 1.12 (0.83-1.52) | .447 |
| 85+ years | 2,8 | 3,0 | 2,4 | 1.26 (0.96-1.65) | .090 |
| Unknown | **0,7** | **0,7** | **0,7** | **0.93 (0.74-1.18)** | **.030** |
|  |  |  |  |  |  |

**Supplementary table I**. Level of Consciousness upon admission for men and women by Age Groups (N=38399).

**Significant sex difference (p < 0.05), CI = Confidence Interval, RR = Relative Risk for women vs. men*

**Supplementary table IIa.** FAST symptoms for men and women by Age Groups.

| Focal Deficits | Total (%) | Women (%) | Men (%) | RR (95% CI) | p-value |
| --- | --- | --- | --- | --- | --- |
| Aphasia (N=38398) | **28,6** | **31,2** | **26,6** | **1.17 (1.13-1.21)** | **<.001*** |
| 18-44 years | 21,7 | 21,4 | 21,9 | 0.98 (0.77-1.25) | .168 |
| 45-54 years | 22,3 | 22,1 | 22,5 | 0.98 (0.83-1.16) | .233 |
| 55-64 years | 22,6 | 23,4 | 22,2 | 1.05 (0.94-1.18) | .904 |
| 65-74 years | 25,4 | 27,8 | 24,0 | 1.16 (1.08-1.24) | <.001* |
| 75-84 years | 30,1 | 31,3 | 28,9 | 1.08 (1.03-1.14) | .003* |
| 85+ years | 36,3 | 37,8 | 34,0 | 1.11 (1.05-1.18) | <.001* |
| Arm paresis (N=38399) | **42,2** | **44,7** | **40,1** | **1.11 (1.08-1.14)** | **<.001*** |
| 18-44 years | 36,8 | 39,4 | 34,7 | 1.13 (0.96-1.34) | .133 |
| 45-54 years | 35,8 | 36,2 | 35,5 | 1.02 (0.91-1.15) | .333 |
| 55-64 years | 38,1 | 41,6 | 36,6 | 1.14 (1.06-1.23) | <.001* |
| 65-74 years | 39,9 | 41,1 | 39,2 | 1.04 (1.00-1.10) | .066 |
| 75-84 years | 42,1 | 43,2 | 41,3 | 1.05 (1.00-1.09) | 0.03* |
| 85+ years | 49,4 | 51,5 | 46,2 | 1.12 (1.07-1.17) | <.001* |
|  |  |  |  |  |  |
| Dysarthria (N=38399) | **29,3** | **29,3** | **29,4** | **1.00 (0.97-1.03)** | **.870** |
| 18-44 years | 19,3 | 19,6 | 19,1 | 1.02 (0.79-1.33) | .854 |
| 45-54 years | 24,7 | 22,1 | 26,1 | 0.84 (0.72-0.99) | .039* |
| 55-64 years | 26,7 | 23,2 | 28,3 | 0.82 (0.73-0.91) | <.001* |
| 65-74 years | 27,8 | 26,9 | 28,3 | 0.95 (0.89-1.02) | .153 |
| 75-84 years | 29,9 | 29,6 | 30,1 | 0.99 (0.93-1.04) | .619 |
| 85+ years | 34,3 | 34,3 | 34,4 | 1.00 (0.94-1.06) | .934 |
|  |  |  |  |  |  |
| Facial Palsy (N=38399) | **38,2** | **39,9** | **37,0** | **1.08 (1.05-1.11)** | **<.001*** |
| 18-44 years | 27,8 | 30,0 | 26,1 | 1.15 (0.94-1.40) | .181 |
| 45-54 years | 31,2 | 30,7 | 31,5 | 0.98 (0.86-1.11) | .721 |
| 55-64 years | 34,1 | 34,9 | 33,7 | 1.03 (0.95-1.26) | .434 |
| 65-74 years | 36,1 | 37,2 | 35,4 | 1.05 (1.00-1.11) | .067 |
| 75-84 years | 39,1 | 39,2 | 39,0 | 1.01 (0.96-1.05) | .822 |
| 85+ years | 45,0 | 46,2 | 43,3 | 1.07 (1.02-1.12) | .011* |

**Significant sex difference (p < 0.05), CI = Confidence Interval, RR = Relative Risk for women vs. men*

**Supplementary table IIb**. Other Focal Deficits for men and women by Age Groups.

| Focal Deficits | Total (%) | Women (%) | Men (%) | RR (95% CI) | p-value |
| --- | --- | --- | --- | --- | --- |
| Leg Paresis (N=38399) | **38,0** | **41,5** | **35,1** | **1.18 (1.15-1.21)** | **<.001*** |
| 18-44 years | 31,0 | 31,6 | 30,5 | 1.03 (0.86-1.25) | .723 |
| 45-54 years | 30,0 | 30,9 | 29,5 | 1.05 (0.92-1.20) | .506 |
| 55-64 years | 33,5 | 37,2 | 31,9 | 1.17 (1.07-1.27) | <.001* |
| 65-74 years | 34,4 | 37,1 | 32,8 | 1.13 (1.07-1.20) | <.001* |
| 75-84 years | 38,7 | 40,9 | 36,8 | 1.11 (1.06-1.16) | <.001* |
| 85+ years | 46,5 | 49,0 | 42,6 | 1.15 (1.09-1.21) | <.001* |
|  |  |  |  |  |  |
| Sensory Deficits (N=38399) | **20,3** | **19,9** | **20,6** | **0.96 (0.93-1.00)** | **.073** |
| 18-44 years | 32,7 | 36,4 | 29,8 | 1.22 (1.02-1.46) | .028* |
| 45-54 years | 29,3 | 31,0 | 28,4 | 1.09 (0.95-1.25) | .203 |
| 55-64 years | 26,1 | 25,1 | 26,5 | 0.95 (0.85-1.05) | .304 |
| 65-74 years | 21,1 | 21,0 | 21,2 | 0.97 (0.90-1.05 | .507 |
| 75-84 years | 17,6 | 18,1 | 17,1 | 1.06 (0.98-1.14) | .155 |
| 85+ years | 16,2 | 16,5 | 15,6 | 1.06 (0.95-1.17) | .293 |
|  |  |  |  |  |  |
| Ataxia (N=38399) | **16,0** | **14,5** | **17,1** | **0.85 (0.81-0.89)** | **<.001*** |
| 18-44 years | 12,2 | 11,1 | 13,1 | 0.85 (0.60-1.20) | .345 |
| 45-54 years | 14,4 | 13,0 | 15,1 | 0.86 (0.69-1.07) | .186 |
| 55-64 years | 15,7 | 14,4 | 16,3 | 0.88 (0.76-1.02) | .095 |
| 65-74 years | 16,6 | 15,7 | 17,2 | 0.91 (0.83-1.00) | .058 |
| 75-84 years | 16,9 | 15,5 | 18,0 | 0.86 (0.79-0.93) | <.001* |
| 85+ years | 14,9 | 13,2 | 17,5 | 0.75 (0.68-0.84) | <.001* |
|  |  |  |  |  |  |
| Visual Field Deficits (N=38399) | **14,0** | **14,2** | **13,9** | **1.02 (0.97-1.07)** | **.408** |
| 18-44 years | 11,3 | 12,0 | 10,8 | 1.10 (0.78-1.57) | .578 |
| 45-54 years | 11,0 | 10,6 | 11,2 | 0.94 (0.73-1.21) | .626 |
| 55-64 years | 12,4 | 11,8 | 12,6 | 0.93 (0.79-1.10) | .400 |
| 65-74 years | 13,8 | 12,9 | 14,3 | 0.90 (0.81-1.00) | .057 |
| 75-84 years | 14,6 | 14,9 | 14,4 | 1.04 (0.95-1.13) | .413 |
| 85+ years | 15,3 | 15,6 | 14,9 | 1.05 (0.95-1.16) | .371 |
|  |  |  |  |  |  |
| Neglect (N=38399) | **11,8** | **13,2** | **10,6** | **1.24 (1.18-1.31)** | **<.001*** |
| 18-44 years | 5,7 | 4,8 | 6,4 | 0.75 (0.44-1.27) | .289 |
| 45-54 years | 6,6 | 7,0 | 6,4 | 1.08 (0.78-1.50) | .625 |
| 55-64 years | 8,3 | 8,3 | 8,2 | 1.01 (0.82-1.24) | .950 |
| 65-74 years | 9,9 | 10,1 | 9,7 | 1.05 (0.93-1.19) | .459 |
| 75-84 years | 12,6 | 13,7 | 11,7 | 1.18 (1.07-1.29) | <.001* |
| 85+ years | 16,8 | 17,9 | 15,3 | 1.17 (1.06-1.29) | .002* |
|  |  |  |  |  |  |
| Vertigo (N=38399) | **8,9** | **8,7** | **9,2** | **0.95 (0.89-1.01)** | **.100** |
| 18-44 years | 15,8 | 14,1 | 17,3 | 0.81 (0.60-1.09) | .172 |
| 45-54 years | 11,7 | 11,4 | 11,9 | 0.95 (0.75-1.22) | .696 |
| 55-64 years | 10,8 | 10,7 | 10,9 | 0.99 (0.83-1.18) | .915 |
| 65-74 years | 9,9 | 9,8 | 9,9 | 0.99 (0.87-1.12) | .820 |
| 75-84 years | 8,5 | 9,4 | 7,8 | 1.19 (1.06-1.34) | .003* |
| 85+ years | 5,8 | 5,7 | 6,1 | 0.93 (0.78-1.10) | .395 |
|  |  |  |  |  |  |
| Dysphagia (N=30833) | **3,5** | **3,6** | **3,4** | **1.06 (0.94-1.20)** | **.308** |
| 18-44 years | 1,3 | 0,6 | 1,8 | 0.32 (0.07-1.48) | .144 |
| 45-54 years | 2,1 | 2,1 | 2,1 | 1.00 (0.51-1.94) | .989 |
| 55-64 years | 2,5 | 2,8 | 2,4 | 1.15 (0.76-1.75) | .499 |
| 65-74 years | 3,0 | 2,6 | 3,2 | 0.79 (0.61-1.04) | .098 |
| 75-84 years | 3,8 | 3,7 | 3,8 | 0.98 (0.81-1.20) | .878 |
| 85+ years | 4,7 | 4,9 | 4,5 | 1.08 (0.86-1.35) | .516 |
|  |  |  |  |  |  |
| Double Vision (N=38399) | **3,2** | **2,6** | **3,7** | **0.72 (0.64-0.81)** | **<.001*** |
| 18-44 years | 4,6 | 3,9 | 5,1 | 0.76 (0.42-1.37) | .364 |
| 45-54 years | 4,0 | 3,1 | 4,4 | 0.69 (0.43-1.11) | .126 |
| 55-64 years | 4,1 | 3,9 | 4,2 | 0.91 (0.68-1.24) | .558 |
| 65-74 years | 3,6 | 3,0 | 4,0 | 0.76 (0.61-0.96) | .018* |
| 75-84 years | 3,0 | 2,6 | 3,4 | 0.75 (0.61-0.92) | .005* |
| 85+ years | 2,1 | 1,9 | 2,4 | 0.81 (0.60-1.08) | .152 |

**Significant sex difference (p<0,05), CI=Confidence Interval, RR=Relative Risk for women vs. Men.*

|  | Women | | Men | |  |
| --- | --- | --- | --- | --- | --- |
| Time Intervals | **Mean (SD)** | **Median** | **Mean (SD)** | **Median** | **p-value** |
| Symptom onset to EMCC notification (N=18744) | **298,34 (460,08)** | **94** | **282,27 (444,38)** | **85** | **.015*** |
| 18-44 years | 184,43 (342,44) | 38,5 | 221,21 (368,89) | 56 | .288 |
| 45-54 years | 258,46 (426,97) | 56,5 | 269,98 (441,25) | 75 | .701 |
| 55-64 years | 310,73 (506,52) | 83 | 293,57(477,04) | 80 | .450 |
| 65-74 years | 312,95 (498,99) | 93 | 276,70 (443,71) | 76,5 | .013* |
| 75-84 years | 304,66 (460,04) | 101 | 282,32 (430,25) | 86 | .051 |
| 85+ years | 293,61 (435,44) | 104 | 293,49 (451,47) | 101 | .993 |
|  |  |  |  |  |  |
| EMCC notification to hospital admission (N=18887) | **63,34 (48,61)** | **50** | **64,69 (49,28)** | **51** | **.059** |
| 18-44 years | 54,06 (40,77) | 42 | 58,52 (51,23) | 45 | .324 |
| 45-54 years | 60,94 (50,80) | 50 | 58,34 (38,49) | 47,5 | .375 |
| 55-64 years | 60,08 (49,92) | 47 | 62,38 (48,59) | 48 | .311 |
| 65-74 years | 62,09 (46,84) | 50 | 64,54 (49,43) | 51 | .106 |
| 75-84 years | 64,62 (49,67) | 51 | 66,51 (49,85) | 53 | .138 |
| 85+ years | 64,44 (48,38) | 51 | 66,64 (51,41) | 53 | .136 |
|  |  |  |  |  |  |
| Hospital admission to cerebral CT/MRI (N=25432) | **121,21 (481,74)** | **28** | **124,53 (557,95)** | **27** | **.616** |
| 18-44 years | 92,08 (204,09) | 21 | 114,39 (287,55) | 24 | .262 |
| 45-54 years | 117,06 (493,16) | 24 | 152,39 (865,02) | 23 | .403 |
| 55-64 years | 126,47 (409,97) | 28 | 141,33 (585,96) | 27 | .539 |
| 65-74 years | 143,73 (746,13) | 29 | 119,30 (516,60) | 26 | .126 |
| 75-84 years | 121,05 (449,37) | 27 | 111,83 (420,50) | 29 | .341 |
| 85+ years | 108,49 (292,55) | 29 | 131,78 (585,24) | 28 | .045* |
|  |  |  |  |  |  |
| Hospital admission to thrombolysis (N=7372) | **36,65 (23,74)** | **30** | **35,60 (25,07)** | **28** | **.067** |
| 18-44 years | 37,02 (21,35) | 31 | 35,73 (24,41) | 30 | .650 |
| 45-54 years | 36,53 (23,83) | 30 | 33,74 (22,37) | 28 | .186 |
| 55-64 years | 37,08 (23,88) | 30 | 38,13 (27,73) | 30 | .555 |
| 65-74 years | 38,18 (26,49) | 31 | 35,51 (25,92) | 27,5 | .030* |
| 75-84 years | 34,84 (22,30) | 28 | 34,85 (24,07) | 28 | .994 |
| 85+ years | 37,44 (23,23) | 31 | 35,13 (22,96) | 29 | .072 |
|  |  |  |  |  |  |
| Hospital admission to thrombectomy (N=1734) | **180,30 (175,29)** | **135** | **184,88 (199,18)** | **135** | **.614** |
| 18-44 years | 173,40 (277,52) | 103,5 | 201,64 (229,41) | 108 | .639 |
| 45-54 years | 152,09 (162,12) | 105 | 207,75 (256,48) | 140 | .157 |
| 55-64 years | 190,93 (210,63) | 148,5 | 201,57 (248,21) | 135 | .726 |
| 65-74 years | 206,43 (178,19) | 178 | 184,26 (175,58) | 143 | .190 |
| 75-84 years | 179,70 (167,95) | 130 | 177,09 (172,03) | 132 | .857 |
| 85+ years | 158,04 (138,10) | 115 | 150,34 (164,20) | 116 | .693 |

**Supplementary table III.** Time Intervals of the Cerebral Stroke Treatment chain in minutes for men and women by Age Groups.

*SD: Standard Deviation, *Significant sex difference (p<0.05), time in minutes*
